# Progress in State Medicine

**Published:** 1898-01-22

**Authors:** 


### PROGRESS IN STATE MEDICINE.

ARTIFICIAL LIGHTING.

Haldane[1] draws attention to the dangerous practice of mixing water-gas with ordinary coal-gas. The mixture as supplied to houses in many of our large towns contains 18 to 20 per cent. of carbonic oxide, thus being three or four times as poisonous as it was in former times. Water-gas is added for the sake of economy, it being quickly and cheaply manufactured, and having a fair illuminating power. Persons accidentally breathing an atmosphere containing these gases would be exposed to great danger. Supposing the atmosphere were to contain ½ per cent. of the mixture, then if the gases contained 20 per cent. of cabonic oxide, this would be sufficient to cause complete helplessness; and if the amount were to reach 1 per cent., death would ensue. It is a matter for very serious consideration whether legislative restrictions should not be placed on the public supply of lighting gas containing more than a moderate percentage of carbonic acid. In America, the addition of carburetted water-gas is even more common than in this country, and the deaths from poisoning by illuminating gas are correspondingly more frequent. Cattell[2] gives a list of 33 cases in Philadelphia; whilst 29 deaths from this cause were reported in Boston during 1895, and 43 during 1896. Carbonic oxide is the only really active poisonous constituent of coal-gas, and although its combination with hæmoglobin is a very close one, it is undoubtedly capable of dissociation. The most satisfactory course of treatment is by means of oxygen under pressure.

Dargelos,[3] attributing various troubles amongst school children, such as conjunctivitis, headache, &c., to the effect of direct bright lights, recommends the use of reflected light only. Copper reflectors, silvered on their upper surface, are placed beneath incandescent burners, so as to throw the light on the ceiling, from whence it was uniformly reflected, giving good illumination and a freedom from shadows. The application of the same principle is advocated by Kermauner and Prausnitz[4] for use in lecture-rooms, workshops, &c. The light is reflected by an opal glass shade beneath the burner, and the jets arranged so as to secure a uniform distribution of light and radiant heat. The lamps are suspended 3 ft. from the ceiling, or about 6 ft. above the table, one burner and inverted reflector being required for every 132 sq. ft. of surface. It is claimed that lighting by this method ensures higher local illumination, a lower temperature, freedom from shadows, and a considerable economy. The data provided have been obtained from experiments in rooms with distempered or whitewashed walls, and it would seem certain that no such good result could be obtained from a dully-papered surface.

**Food and Drugs.**—The fact that it was found necessary during the last session of Parliament to drop the Food and Drugs Amendment Bill is a matter for regret.[5] Amongst the proposed changes were several of great importance. The terms were to include articles "intended to enter into or be used in the preparation of human food, and flavouring matters and condiments." The scale of penalties had been revised, and it was proposed that frequent offences should be visited by the publication of the name of the offender. The procedure in defence of warranty was amended, and inspectors were given the power of visiting, and of taking samples from, the manufacturer and wholesale dealer. All public authorities to submit for analysis a minimum number of samples annually at the rate of one to every thousand inhabitants. A central board of reference was to help in the further administering of the Act, the determining of standards, &c.

The addition of colouring materials to food,[6] provided such materials are free from metallic impurities, is not as a rule prejudicial to health. Coal-tar colours are those most commonly employed, for they are very soluble, and have a high colouring value. Of the yellow pigment sometimes added to butter much less than a grain is eaten at an ordinary meal, whilst of the colouring materials of jams and jellies the amount is such that several ounces of the food would have to be eaten to include one-third of a grain of the colour.

Such minute quantities would not interfere with the action of the digestive agents, nor is any toxic action likely to follow their use. The use of metallic colours, such as the salts of copper, is a much more objectionable practice. Opinions are divided as to whether cases of poisoning have been traced to this source, but in spite of this, penalties have been inflicted upon persons found guilty of retailing bottled peas so coloured. In testing, if the food be not fluid, macerate in warm distilled water. If the colouring matter be readily taken up by the water, and the colour be bleached by solution of hypochlorite of sodium, it is organic and probably harmless; if not, the colouring matter should be dissolved out by acids and the solution tested for metals.

The addition of chemical substances to foods in order to secure their preservation is not a punishable offence, unless the procedure can be proved to be injurious to health. Thus boracic acid and formalin are added to milk; salicylic and benzoic acids to beer, cider, and wines. Bacon, sausages, and fish frequently are treated with borax to ensure their keeping, similarly salicylic acid is used in the bottling of fruit, and salts of zinc or lead added to cheese to prevent heaving and cracking. A special sanitary commission of the *Lancet*[7] recently obtained the opinions of a large number of experts regarding the injuriousness of such adulteration. The opinion was very generally expressed that the ingestion of small quantities of such preservatives was not likely to give rise to toxic symptoms, but at the same time it was essential that a stop should be put to this growing practice of sophistication, and the Commission recommended that whenever any foreign substance was added to a food the name and quantity of the material employed should be stated, either by affixing a label or by other means. There are many reasons why some such procedure is necessary. The addition of these mild antiseptics may give to the food a false freshness, for the preserved articles may be in a state of incipient decomposition.[8] Again, it seems probable that boric acid may precipitate and render insoluble certain salts which give to milk its high nutritive value, and, furthermore, may so affect the gastric juice as to retard digestion. Such milk would be especially prejudicial to infants. Some people, too, have an idiosyncrasy for certain drugs, and might be injuriously affected by even small doses; thus, it is important to keep the quantity as low as possible.

British wines frequently contain 2 to 3 grains of salicylic or benzoic acid per pint, the syrups of preserved fruits 20 grains per pint, whilst fish, bacon, and sausages have sometimes higher percentages of boric acid. In a case recently tried at Glamorgan, Maclay[9] reports that the sample of milk was found to contain 52 grains per quart, or three-tenths per cent. of adulteration. It is noteworthy that the prosecution was successful. One-tenth is probably the maximum that should be allowed. If the addition of preservatives is to be countenanced (and it is difficult to see how the practice can be checked), it is essential that some standard shall be established, and that the fact of such an addition shall be notified to the purchaser.

[1] Brit. Med. Jour., Oct. 3, 1896. [2] Internat. Medical Magaz. [3] Annales d'Hygiene, XXXV. [4] Archiv. für Hyg., XXIX., 2. [5] Practitioner, July, 1897. [6] The Dietetic and Hygienic Gazette, March. 1897. [7] Lancet, Jan. 2, 1897. [8] Pacific Medical Journal, July, 1896. [9] Sanitary Record, Sept. 17, 1897.

